# Kokain-induzierte Vaskulitiden und Vaskulitis-Mimics

**DOI:** 10.1007/s00393-022-01217-1

**Published:** 2022-05-25

**Authors:** Nikolas Ruffer, Martin Krusche, Konstanze Holl-Ulrich, Ina Kötter, Fabian Lötscher

**Affiliations:** 1 Klinik für Rheumatologie und Immunologie, Klinikum Bad Bramstedt, Bad Bramstedt, Deutschland; 2 Sektion Rheumatologie und entzündliche Systemerkrankungen, III. Medizinische Klinik und Poliklinik, Universitätsklinikum Hamburg-Eppendorf, Martinistr. 52, 20246 Hamburg, Deutschland; 3 Konsultations- und Referenzzentrum für Vaskulitis-Diagnostik, Pathologie-Hamburg, Labor Lademannbogen MVZ GmbH, Hamburg, Deutschland; 4 Universitätsklinik für Rheumatologie, Immunologie und Allergologie, Inselspital, Universitätsspital Bern, Bern, Schweiz

**Keywords:** Cocaine-induced midline destructive lesions, Levamisol, Granulomatose mit Polyangiitis, ANCA, Mimics, Cocaine-induced midline destructive lesions, Levamisole, Granulomatosis with polyangiitis, ANCA, Mimics

## Abstract

Kokain ist ein psychoaktives Tropanalkaloid, das typischerweise in Form von Pulver nasal konsumiert wird. Das in Deutschland im Umlauf befindliche Kokain ist häufig mit Levamisol, einem Anthelminthikum mit immunmodulatorischer Wirkung, versetzt. Sowohl Kokain als auch Levamisol werden mit der Entwicklung von klinisch bedeutsamen Autoimmunphänomenen in Verbindung gebracht. Bei den sog. „cocaine-induced midline destructive lesions“ handelt es sich um Gewebedestruktionen des HNO-Traktes, die eine lokalisierte Granulomatose mit Polyangiitis imitieren können. Zusätzlich wurde auch die Entwicklung einer systemischen Vaskulitis durch Kokain und Levamisol beschrieben. Die Unterscheidung dieser Syndrome von einer primären Vaskulitis stellt häufig eine klinische Herausforderung dar, da immunserologisch in den meisten Fällen antineutrophile zytoplasmatische Antikörper (ANCA) nachweisbar sind. Die klinische Besserung ist eng mit der Beendigung des Substanzkonsums verknüpft, deshalb kommt der frühzeitigen Diagnosestellung eine besondere Bedeutung zu.

## Abstract (en)

Cocaine is a psychotropic tropane alkaloid and stimulant drug. Nasal insufflation of cocaine powder is a common route of administration. In Germany, cocaine is frequently adulterated with levamisole, an anthelminthic drug with immunomodulatory effects. Both substances are linked to various autoimmune conditions. Cocaine-induced midline destructive lesions cause a progressive destruction of osteocartilaginous structures within the upper respiratory tract and can mimic localized granulomatosis with polyangiitis. In addition, systemic vasculitis due to cocaine and levamisole has been reported. Differentiation of these conditions from primary vasculitis can be challenging because antineutrophil cytoplasmic antibodies (ANCA) are commonly detected. Early diagnosis of these conditions is crucial as clinical improvement is closely related to drug cessation.

Kokain^1^ (Benzoylecgoninmethylester) ist ein psychoaktives Tropanalkaloid, das aus den Blättern des Kokastrauches (Pflanzengattung: *Erythroxylon*) gewonnen wird. Es ist nach Cannabis die zweithäufigste illegale Rauschdroge, die in der Europäischen Union (EU) konsumiert wird [2]. Die Europäische Beobachtungsstelle für Drogen und Drogensucht (EMCDDA) bezifferte 2018 die Lebenszeitprävalenz des Kokainkonsums für 15- bis 65-Jährige in Deutschland mit 4,1 % [3].

In Abhängigkeit von der Darreichungsform kann Kokain inhaliert, injiziert oder lokal über die Schleimhäute aufgenommen werden. Die nasale Aufnahme („schnupfen“) als weißes Pulver (Salzverbindung Kokain-HCl) mit Resorption über die Nasenschleimhäute ist weit verbreitet. Zusätzlich kann es durch Weiterverarbeitung zu „Crack“ geraucht oder auch parenteral als Lösung konsumiert werden. Insbesondere in Südamerika wird auch das Kauen von Kokablättern praktiziert.

Als Monoamin-Wiederaufnahmehemmer hat Kokain kurzfristig eine euphorisierende und antriebssteigernde Wirkung. Daneben können sich jedoch auch verschiedenste psychiatrische (z. B. Psychose), neurologische (z. B. Kopfschmerzen) und internistische Folgeschäden (z. B. Myokardinfarkt) entwickeln [4].

Ein Großteil des in Deutschland konsumierten Kokains ist mit Levamisol, ein Derivat der Imidazothiazole mit immunmodulatorischer Wirkung, versetzt [5]. Sowohl Kokain als auch Levamisol werden mit der Entwicklung von klinisch bedeutsamen Autoimmunphänomenen in Verbindung gebracht [6]. Unter anderem ist die Bildung von antineutrophilen zytoplasmatischen Antikörpern (ANCA) beschrieben worden [7–9].

Aus rheumatologischer Sicht sind v. a. zwei klinische Syndrome von Bedeutung, da sie eine primäre ANCA-assoziierte Vaskulitis (AAV) imitieren^2^ können („mimics“) [11]: Die sog. *„cocaine-induced midline destructive lesions“* (CIMDL) können den klinischen Phänotyp einer lokalisierten Granulomatose mit Polyangiitis (GPA) nachahmen [12]. Typische Befunde weiterer Organmanifestationen einer GPA (z. B. Glomerulonephritis) sind in diesen Fällen jedoch sehr selten. Im Gegensatz hierzu ist auch die Entwicklung eines Vaskulitissyndroms mit systemischen Manifestationen durch Kokain bzw. Levamisol als *Kokain/Levamisol-induzierte Vaskulitis* (CLIV) beschrieben worden [13–15]. Als charakteristisch für Levamisol-induzierte Vaskulitissyndrome gelten die Befunde einer Neutropenie und retiformen Purpura insbesondere im Bereich der Ohren [13, 16, 17].

Die Unterscheidung dieser Syndrome von einer primären Vaskulitis stellt in vielen Fällen eine Herausforderung dar, weil sich in der Autoimmunserologie sehr häufig ANCA nachweisen lassen. Da eine klinische Besserung eng mit der Beendigung des Substanzkonsums verknüpft ist, kommt der frühzeitigen Diagnosestellung eine besondere Bedeutung zu. Fälle mit einer verspäteten Diagnosestellung („therapierefraktäre GPA“) sind möglicherweise ohne nachhaltigen Therapieerfolg mit Glukokortikoiden und potenten *disease-modifying anti-rheumatic drugs* (DMARD) behandelt worden [15, 18].

Bereits 1910 erkannte der Militärarzt W.D. Owens [19] ulzerierende Läsionen der Nasenschleimhaut als klinisches Zeichen des Kokainkonsums. Ausgeprägte Mittelgesichtsdestruktionen durch Kokain sind jedoch erst in den 1980er-Jahren beschrieben worden [20]. Die Erstbeschreibungen von Kokain-assoziierten Vaskulitissyndromen stammen auch aus dieser Zeit [21]. Kaye und Fainstat [22] berichteten 1987 wiederum erstmalig von einer isolierten zerebralen Vaskulitis. Enríquez et al. [23] beschrieben 1991 den Befund einer leukozytoklastischen Vaskulitis in Zusammenhang mit dem Konsum von Kokain. Den ersten Fallbericht einer Levamisol-induzierten Vaskulitis lieferten Macfarlane et al. [24] im Jahr 1978.

## „Cocaine-induced midline destructive lesions“ (CIMDL)

**Figure 1:**
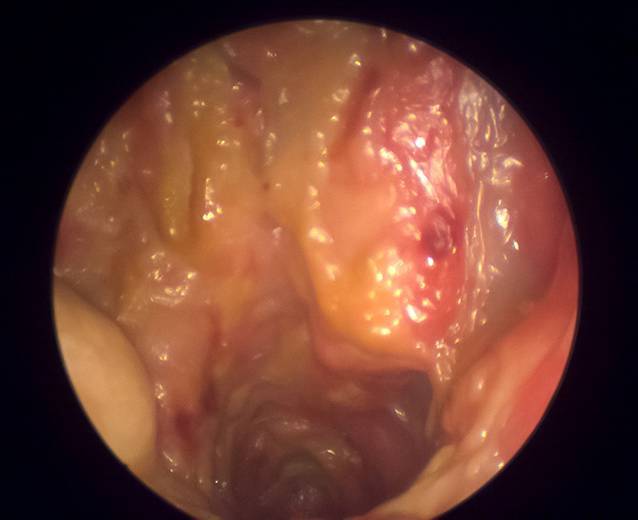

Als CIMDL werden entzündlich destruierende Veränderungen des HNO-Traktes bezeichnet, die durch nasalen Kokainkonsum entstehen [12]. Während initial v. a. die nasale Mukosa betroffen ist, können bei chronischem Konsum auch ausgedehnte Schäden an den Knorpel- und Knochenstrukturen im HNO-Trakt und Mittelgesicht entstehen [25]. Klinisch bestehen häufig eine nasale Obstruktion mit Hyposmie, Epistaxis mit Krustenabgang und Gesichtsschmerzen [11, 25]. Grundlage hierfür sind meistens chronische Sinusitiden. Bei der Inspektion und endoskopischen Untersuchung zeigen sich nekrotische und ulzerierende Läsionen, häufig mit Krustenbildung (Abb. 1; [11, 25]). Klinisch kann auch eine Sattelnase vorliegen. Selten können sich sogar Läsionen im Bereich der Orbita und Schädelbasis entwickeln [15, 26, 27]. Auch zerebrale Läsionen sind in Extremfällen beschrieben worden [27, 28].

**Figure 2:**
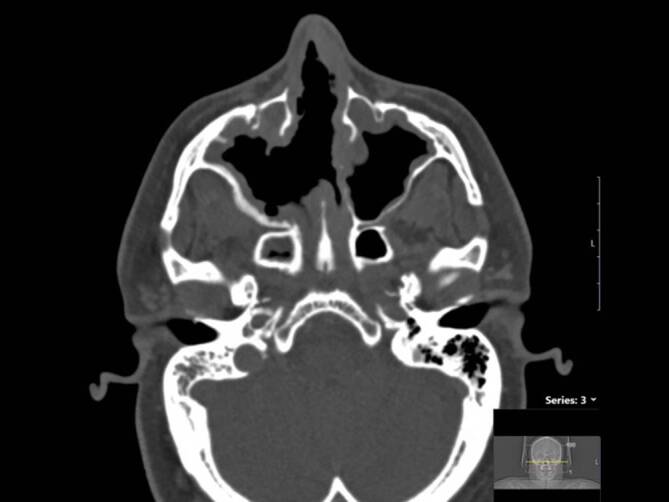

In der bisher größten Fallserie von CIMDL (25 Fälle) zeigte sich bei allen Fällen eine Nasenseptumperforation (Abb. 2), und in etwa zwei Drittel der Fälle (68 %) bestand eine Destruktion der Conchae nasales inferiores [29]. Die Conchae nasales superiores waren seltener (16 %) betroffen [29]. Gaumenperforationen fanden sich bei etwa einem Viertel der Fälle (24 %) [29]. Eine vollständige Destruktion des lateralen Nasenskelettes fand sich in 20 % der Fälle [29]. Eine Affektion der Columella und Oberlippe sind auch beschrieben worden und gelten wiederum als untypisch für eine GPA [30].

### Autoimmunserologie

**Figure 3:**
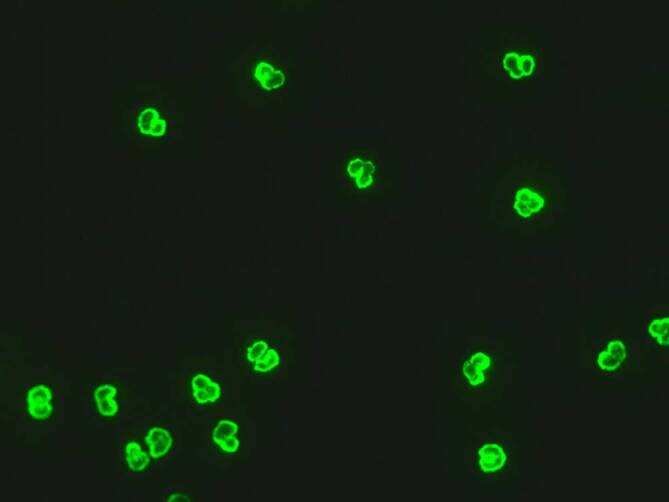

ANCA können in der Mehrheit der Fälle nachgewiesen werden, weshalb zur weiteren Einordnung des Befundes neben den initial zu bestimmenden antigenspezifischen ELISA zwingend auch die Immunfluoreszenz beurteilt werden muss [12, 31]. In der Immunfluoreszenz zeigt sich meistens (bis zu 72 % der Fälle) ein perinukleäres Muster (p-ANCA) (Abb. 3; [7, 12]). Hierbei handelt es sich in bis zu 84 % der Fälle um Antikörper gegen humane Leukozytenelastase^3^ (HLE-ANCA), eine Serinprotease [7, 32]. Interessanterweise finden sich in einigen Fällen zusätzlich auch Antikörper gegen Proteinase‑3 (PR3-ANCA) [12]. Antikörper gegen Myeloperoxidase (MPO-ANCA) sind bei CIMDL bisher nicht beschrieben worden [7, 29]. Die konsistente Kombination aus zytoplasmatischem Muster (c-ANCA) mit Nachweis von Antikörpern gegen PR3 ist jedoch ungewöhnlich für CIMDL und deutet auf eine GPA hin.

### Histologische Befunde

Die histologische Untersuchung von HNO-Biopsaten bei CIMDL zeigt unspezifische Befunde, die auch bei der GPA auftreten können [32].

**Figure 4:**
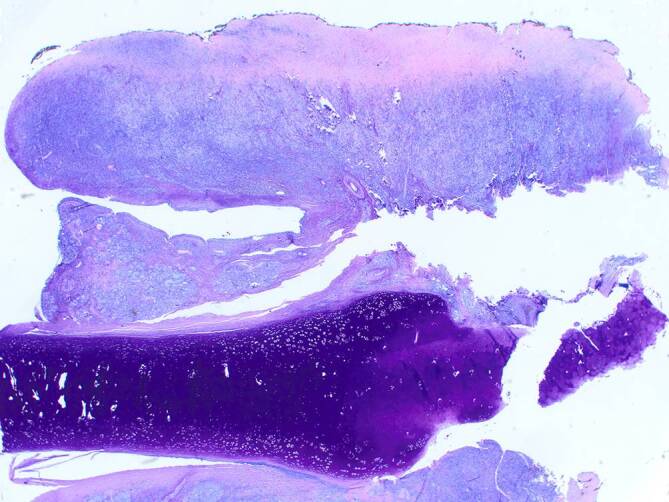

Bei CIMDL können fibrotische Veränderungen und zum Teil ausgedehnte nekrotische Läsionen (Abb. 4) nachgewiesen werden [29, 32]. Zusätzlich kann auch eine entzündliche Infiltration von Venolen und Arteriolen („Perivenulitis“) bestehen [29, 32]. Ebenfalls beschrieben sind Mikroabszesse in Gefäßwänden, thrombotische Veränderungen und Befunde einer Vaskulitis mit fibrinoider Nekrose [29, 32]. Eine sichere histologische Unterscheidung zwischen CIMDL und GPA gelingt daher nur durch den Nachweis von spezifischen (extravaskulären) GPA-Läsionen (s. unten) [12].

Im Gegensatz zu den CIMDL ist die GPA typischerweise durch den histologischen Nachweis von (1) unscharf begrenzten epitheloidzelligen Granulomen mit extravaskulärer Lokalisation, (2) landkartenartigen Nekrosen mit histiozytärem Randwall und (3) nekrotisierender Vaskulitis gekennzeichnet [33]. Die beschriebene Trias findet sich jedoch nur in etwa 16 % der Fälle [34]. Bei renaler Beteiligung besteht das histologische Bild einer nekrotisierenden Glomerulonephritis mit extrakapillärer Proliferation.

### Pathogenese

Die Pathogenese von CIMDL ist nicht abschließend geklärt. Diskutiert werden zum einen ischämische Effekte von Kokain (Vasokonstriktion) und eine direkte Traumatisierung durch Kokainkristalle [12]. Zusätzlich besteht fast immer eine bakterielle Superinfektion, welche eine chronische Entzündung verursacht [29]. Inwieweit den ANCA eine pathogenetische Bedeutung zukommt, ist unklar. Experimentelle Daten zeigen jedoch, dass die HLE-ANCA einen inhibitorischen Effekt auf die Enzymaktivität der HLE haben und über diesen Mechanismus nicht an der Pathogenese der CIMDL beteiligt sind [8]. Zusätzlich scheint Kokain bei respiratorischen Epithelien Apoptose-induzierende Effekte zu haben [12].

### Differenzialdiagnose

Die Diagnose von CIMDL basiert im Wesentlichen auf dem klinischen Aspekt von entzündlich destruierenden Läsionen des HNO-Traktes und dem Nachweis von Kokainmetaboliten in Blut oder Urin. Da der Kokainkonsum von den Patient:innen häufig verneint wird und die laboranalytische Bestätigung gerade deshalb in vielen Fällen nicht angestrebt wird, werden die Läsionen häufig als „atypische GPA“ gewertet. „Red flags“ als Hinweis auf CIMDL finden sich in Tab. 1. Vor diesem Hintergrund sollten die wichtigsten Differenzialdiagnosen der CIMDL möglichst ausgeschlossen werden.

**Table 1:** 

*Anamnese*
– Junges Alter
– Bekannter Kokainkonsum
– Fehlendes Ansprechen auf eine potente immunsuppressive Therapie (z. B. Cyclophosphamid oder Rituximab)
*Autoimmunserologie: Nachweis von p‑ANCA bzw. HLE-ANCA*
*Organmanifestationen*
– Perforation des harten Gaumens
– Destruktionen des lateralen Nasenskelettes
– Affektion der Columella
– Affektion der Oberlippe
– Abwesenheit systemischer Manifestationen einer Granulomatose mit Polyangiitis (z. B. pulmonale Granulome)
*Histologie: Fehlen von granulomatösen Veränderungen*

*ANCA* Antineutrophile zytoplasmatische Antikörper, *HLE* Humane Leukozytenelastase

#### Granulomatose mit Polyangiitis.

**Figure 5:**
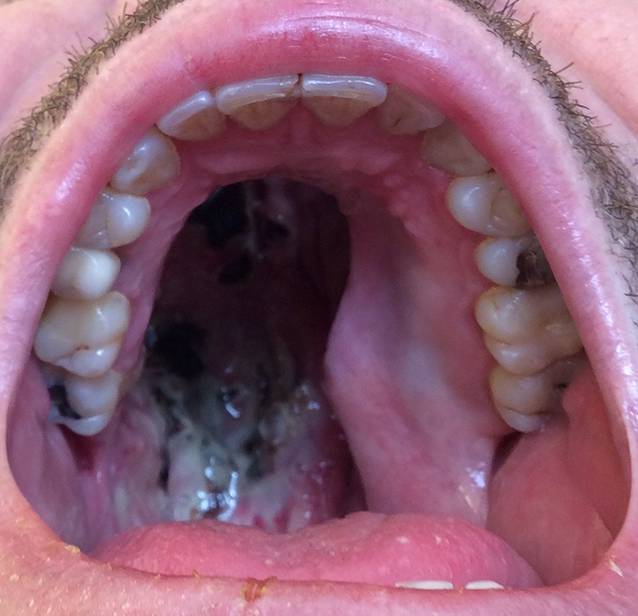

Der häufige Nachweis von ANCA bei CIMDL führt in vielen Fällen zu der Fehldiagnose einer GPA^4^. Die differenzialdiagnostische Abgrenzung basiert auf dem klinischen Bild (systemische Manifestationen?), der Histologie (spezifische GPA-Läsionen?) und der ANCA-Diagnostik (Nachweis von HLE-ANCA?). Klinisch können GPA und CIMDL zu einer Sattelnase führen. Bei der GPA entwickeln sich (im Verlauf) häufig weitere Organmanifestationen, die bei CIMDL in fast allen Fällen fehlen^5^. Problematisch ist, dass HNO-Biopsien auch bei der GPA häufig unspezifische Veränderungen zeigen [34]. Insofern sollte in unklaren Fällen nach systemischen Zeichen einer GPA gefahndet werden (wenn möglich mit histologischer Sicherung) [38]. Mittels Röntgenaufnahme des Thorax sollte beispielsweise nach pulmonalen Manifestationen gesucht werden [38]. Mit der Bestimmung von Nierenfunktionsparametern und der Untersuchung von Urinsediment sollte eine renale Beteiligung abgeklärt werden. Eine Perforation des harten Gaumens (Abb. 5) im Rahmen einer GPA ist eine absolute Rarität und spricht sehr stark gegen diese Diagnose [39]. Auch eine Affektion von Columella und Oberlippe sind untypisch für eine GPA [30]. Insgesamt scheinen CIMDL schwerwiegendere Mittelgesichtsdestruktionen zu verursachen als eine lokale GPA [32]. Der histologische Nachweis der oben genannten Trias spricht wiederum für eine GPA. Insbesondere granulomatöse Veränderungen finden sich bei CIMDL nicht [29, 32]. Auch sollte eine weitere ANCA-Differenzierung in Verdachtsfällen ergänzt werden [38]. Der Nachweis von HLE-ANCA deutet auf CIMDL hin, da diese Antikörper bei der GPA bisher nicht beschrieben wurden und in bis zu 84 % der Fälle bei CIMDL zu finden sind [7]. c‑ANCA mit korrespondierendem Nachweis von PR3-ANCA sind wiederum sehr spezifisch für eine GPA. Verdächtig ist auch ein fehlendes therapeutisches Ansprechen einer „lokalisierten GPA“ auf hoch dosierte Glukokortikoide und potente DMARDs (Rituximab [RTX], Cyclophosphamid [CYC]). Bei der Reevaluation einer „therapierefraktären GPA“ sollte die Bestimmung von Kokainmetaboliten im Urin angestrebt werden, um mögliche CIMDL zu identifizieren.

#### Weitere entzündliche Systemerkrankungen.

Als weitere Differenzialdiagnose kommen seltene Manifestationen einer IgG4-assoziierten Erkrankung mit progredienter Mittelgesichtsdestruktion („IgG4-related midline destructive lesion“) in Betracht [40]. Die Abgrenzung gegenüber der GPA kann erschwert sein, da auch bei der GPA erhöhte IgG4-Spiegel im Serum und IgG4-positive Plasmazellen in HNO-Biopsaten auftreten können [41, 42]. Einzig die Kombination aus storiformer Fibrose und einer Ratio IgG4^+^/IgG^+^-Plasmazellen > 20 % erlaubt eine sichere Differenzierung [42]. Interessanterweise beschreiben Subesinghe et al. [14] in ihrer Fallserie sogar einen Fall mit Kokainkonsum und deutlich vermehrten IgG4-positiven Plasmazellen (> 80 %) in einem Biopsat der Nasenschleimhaut^6^. Ferner sollte auch eine Sarkoidose abgeklärt werden [12, 43].

#### Infektionen.

Chronische Infektionen (insbesondere durch sog. „atypische Erreger“) können ebenfalls zu ausgedehnten Destruktionen des HNO-Traktes führen. Daher sollten Mykobakteriosen, eine Lues und Pilzinfektionen durch serologische, mikrobiologische und ggf. histologische Untersuchungen ausgeschlossen werden. Gleichzeitig findet sich bei CIMDL fast immer eine bakterielle Superinfektion der nekrotischen Mukosa, häufig durch *Staphylococcus aureus*. Ferner können sehr seltene Defekte des *TAP*-Gens („transporter complex associated with antigen presentation“) zu rezidivierenden Infekten durch gramnegative Erreger mit konsekutiver Mittelgesichtsdestruktion führen [10]. In Endemiegebieten sollte auch an eine kutane Leishmaniose gedacht werden [44].

#### Malignome.

Auch maligne Erkrankungen des HNO-Traktes können zu progredienten Destruktionen von Mittellinienstrukturen führen. Neben epithelialen Tumoren ist auch an nasale NK/T-Zell-Lymphome („natural killer-T-cells“) zu denken, die früher als „lethal midline granuloma“ eigeordnet wurden [10].

### Therapie

An erster Stelle steht eine suchtmedizinische Behandlung. Idealerweise erfolgt die Behandlung in einem interdisziplinären Team aus Allgemeinmedizin, HNO-Heilkunde, Psychiatrie und ggf. Mund-Kiefer-Gesichtschirurgie. Die Beendigung des Substanzkonsums ist von großer Bedeutung für den sonst häufig progredienten Verlauf mit zunehmender Destruktion von HNO-Trakt und Mittelgesicht [25]. Für den Einsatz von Glukokortikoiden und DMARDs gibt es wenig Evidenz, weshalb wir uns Trimarchi et al. [25] anschließen und zunächst keine Indikation für diese Therapien sehen. Von HNO-ärztlicher Seite sollten zunächst lokale Therapieversuche mit Débridement von nekrotischem Gewebe sowie regelmäßige Nasenduschen erfolgen. Auch sollten möglicherweise komorbid bestehende Infekte des HNO-Bereichs lokal antibiotisch (ggf. auch systemisch) behandelt werden [12]. Bei einer Perforation des harten Gaumens kann eine Prothesenversorgung notwendig sein [25]. Rekonstruktive Eingriffe sind im Verlauf bei anhaltender Kokainabstinenz möglich [12].

## Kokain/Levamisol-induzierte Vaskulitis (CLIV)

Sowohl Kokain als auch Levamisol sind mit der Entwicklung von systemischen Vaskulitissyndromen in Verbindung gebracht worden. Klinisch ähneln diese Syndrome häufig einer AAV (insbesondere GPA und mikroskopische Polyangiitis).

Bis in die 1990er-Jahre wurde Levamisol u. a. in der Therapie von Malignomen und der rheumatoiden Arthritis eingesetzt [11]. Hierbei wurden gehäuft Vaskulitissyndrome mit Agranulozytose beobachtet, sodass das Levamisol schließlich vom Markt genommen wurde [45]. Neue Aufmerksamkeit erlangte Levamisol zu Beginn der 2000er-Jahre in den Vereinigten Staaten durch vermehrte Fallberichte von Kokainkonsument:innen mit gleichartigen Krankheitsverläufen [11, 45]. Durch Laboranalysen von Kokainproben konnte eine Beimengung von Levamisol bestätigt werden [11]. Pharmakologische Untersuchungen zeigen, dass Levamisol und sein Metabolit Aminorex die psychoaktive Wirkung von Kokain verstärken bzw. ergänzen könnten [46, 47].

**Figure 6:**
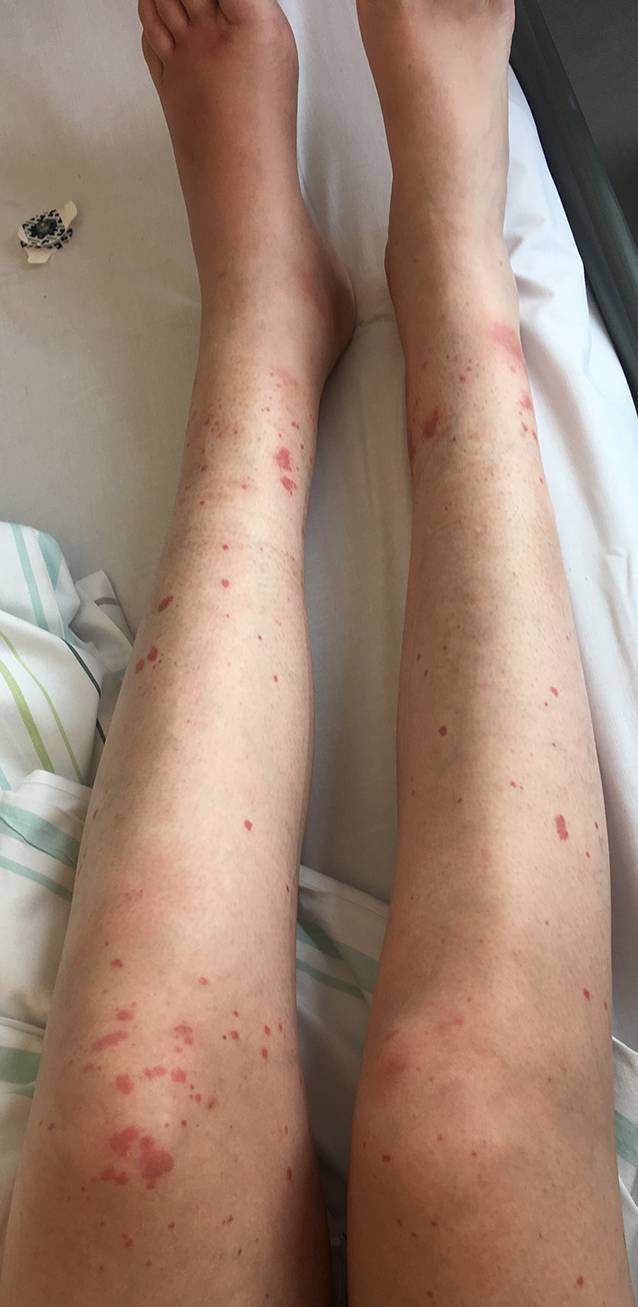

Klinisch besteht bei der CLIV in etwa 80 % der Fälle eine retiforme Purpura, die am häufigsten an der unteren Extremität auftritt (Abb. 6; [13]). Als nahezu pathognomonisch gelten nekrotische Hautveränderungen der Ohren^7^ (v. a. im Bereich der Helix), die bei etwa drei Viertel der Fälle zu beobachten sind [13, 49]. In schweren Fällen können die livedoartigen Läsionen konfluieren und hämorrhagische Blasen bilden [13]. Teilweise entwickeln sich tiefe Nekrosen mit konsekutiver Autoamputation. Zusätzlich besteht ein breites Spektrum extrakutaner Manifestationen. Eine renale (z. B. Glomerulonephritis) und pulmonale Beteiligung (z. B. alveoläre Hämorrhagie) ist ebenfalls möglich, wodurch sich klinische Überlappungen mit einer primären AAV und dem Goodpasture-Syndrom ergeben [15, 50]. In vielen Fällen bestehen auch Beschwerden des Bewegungsapparates in Form von Arthralgien und Myalgien [13]. Das Blutbild zeigt häufig eine Anämie. Eine Neutropenie bzw. Agranulozytose gilt als klassisches Zeichen der Levamisol-Exposition [49].

### Pathogenese

Die Pathogenese der CLIV ist weiterhin Gegenstand der Forschung. Experimentelle Daten von Lood und Hughes [51] deuten jedoch auf die Induktion von NETs („neutrophil extracellular traps“) durch Kokain und Levamisol hin. Spezifische Autoantikörper wie HLE-ANCA können wiederum in NETs enthaltene Autoantigene binden [52]. Zusätzlich könnte Kokain an der Entstehung von autoimmunologisch bedeutsamen Neoantigenen beteiligt sein [52]. Letztendlich kann die Einnahme beider Substanzen zu einem Verlust der immunologischen Toleranz mit systemischer Inflammation führen, die sich klinisch als Vaskulitis manifestiert. Weibliches Geschlecht scheint hierbei ein Risikofaktor zu sein [9].

Auch bei den primären AAV sind NET von pathogenetischer Bedeutung [53, 54]. So konnte beispielsweise gezeigt werden, dass ANCA bei Neutrophilen eine NETose induzieren können [54]. Durch die Freisetzung von proinflammatorischen Mediatoren und Aktivierung des Komplementsystems kommt es letztendlich zu einer Schädigung des Gefäßendothels [54].

### Autoimmunserologie

ANCA können in bis zu 88 % der Fälle einer CLIV nachgewiesen werden. Am häufigsten findet sich dabei ein perinukleäres Muster (p-ANCA) [9, 13, 15, 49]. Typisch ist auch der gleichzeitige Nachweis von PR3-ANCA und MPO-ANCA [9, 13, 15, 49]. Zusätzlich können auch HLE-ANCA bestehen [49]. Ferner finden sich in etwa der Hälfte aller Fälle auch antinukleäre Antikörper (ANA) und Antiphospholipidantikörper [13, 15, 49]. Seltener sind Autoantikörper gegen doppelsträngige DNA (dsDNA) und ein Komplementverbrauch (insbesondere C3) beschrieben [13, 15].

### Histologische Befunde

Die histologische Untersuchung von Hautbiopsaten (Purpura) ergibt in den meisten Fällen den unspezifischen Befund einer leukozytoklastischen Vaskulitis [13, 49]. Daneben sind auch nichtentzündliche Veränderungen im Sinne einer thrombotischen Vaskulopathie beschrieben worden [13, 49]. Granulomatöse Veränderungen fehlen hingegen auch bei der CLIV [14].

Bei renaler Beteiligung bestehen am häufigsten eine pauci-immune nekrotisierende Glomerulonephritis (etwa 50 % der Fälle) und membranöse Glomerulonephritis [15]. Darüber hinaus können jedoch weitere vielfältige Pathologien bestehen, die eine breite Differenzialdiagnostik implizieren (Tab. 2).

**Table 2:** 

Nephropathologisches Befundmuster	Fälle	Prozent (%)
Nicht näher klassifizierbar	2	4,9
Interstitielle Fibrose	1	2,4
C3-assoziierte Glomerulonephritis	1	2,4
Fokal-segmentale Glomerulosklerose	1	2,4
Lupusnephritis (Klasse IV)	1	2,4
Mesangioproliferative/IgA-assoziierte Glomerulonephritis	3	7,3
Immunkomplex-vermittelte Glomerulonephritis	3	7,3
Membranöse Nephropathie	4	9,8
Pauci-immune nekrotisierende Glomerulonephritis	19	46,3
*Begleitende Befundmuster:*		
Pauci-immune nekrotisierende Glomerulonephritis plus	6	14,6
Interstitielle Nephritis	1	2,4
Membranöse Nephropathie	5	12,2
Gesamt	41	100

*C3* Komplementfaktor C3, *IgA* Immunglobulin‑A

In der Lunge ist auch eine pulmonale Vaskulitis (lymphozytäre Vaskulitis) beschrieben worden [50].

### Differenzialdiagnose

Allgemein besteht bei der CLIV das klinische Bild einer entzündlichen Systemerkrankung mit vorwiegender Affektion der Haut. Vor diesem Hintergrund ergibt sich ein breites differenzialdiagnostisches Spektrum. Da große Ähnlichkeiten zu einer primären AAV präsent sein können, sollten diese als Erstes bedacht werden. „Red flags“ als Hinweis auf CLIV finden sich in Tab. 3.

**Table 3:** 

*Anamnese*
– Junges Alter
– Bekannter Kokainkonsum
*Autoimmunserologie*
– Doppelpositivität von PR3-ANCA und MPO-ANCA
– Nachweis von HLE-ANCA
– Nachweis von Antiphospholipidantikörpern
*Organmanifestationen*
– Haut: Befall der Ohren (v. a. Helix), ausgedehnte Hautnekrosen
– Klinischer Phänotyp einer systemischen „Granulomatose mit Polyangiitis“ ohne Beteiligung des HNO-Traktes
– Leukopenie bzw. Neutropenie

*ANCA* Antineutrophile zytoplasmatische Antikörper, *HLE* Humane Leukozytenelastase, *PR3* Proteinase‑3, *MPO* Myeloperoxidase

#### ANCA-assoziierte Vaskulitiden.

In Analogie zu den CIMDL basiert die Abgrenzung von AAV^8^ und CLIV auf Organmanifestationen, Autoimmunserologie und Histologie. Der mitunter ausgeprägte Hautbefall mit konfluierenden Nekrosen ist untypisch für eine AAV [13]. Die Affektion der Ohren (v. a. im Bereich der Helix) gilt als nahezu pathognomonisch für eine CLIV. Im Gegensatz zu einer GPA mit systemischen Manifestationen finden sich bei der CLIV nur selten HNO-Manifestationen [15, 36]. Eine schwere Leukopenie bzw. Neutropenie spricht auch gegen eine AAV, wobei hier auch die Effekte einer möglicherweise bereits initiierten immunsuppressiven Therapie (z. B. CYC oder RTX) der AAV differenzialdiagnostisch berücksichtigt werden müssen. Verdächtig ist auch die Diskrepanz von Immunfluoreszenz und ELISA („enzyme-linked immunosorbent assay“) in der ANCA-Diagnostik (z. B. p‑ANCA und PR3-ANCA). Der gleichzeitige Nachweis von PR3-ANCA und MPO-ANCA gilt ebenfalls als pathognomonisch [13, 15, 49]. Eine weitere Differenzierung kann durch die Bestimmung von HLE-ANCA gelingen [49]. Bei der histologischen Aufarbeitung von Biopsaten sind granulomatöse Veränderungen im Fall einer CLIV nicht nachweisbar [13, 15, 49]. Letztendlich sollte eine „therapierefraktäre AAV“ gerade bei ausgeprägtem Hautbefund bezüglich eines Kokainkonsums untersucht werden.

#### Weitere entzündliche Systemerkrankungen.

Neben einer AAV sind auch pulmorenale Syndrome anderer Genese zu berücksichtigen. Die Bestimmung von Autoantikörpern gegen die glomeruläre Basalmembran (Anti-GBM) und der nephropathologische Befund (lineare Ablagerungen von IgG an der GBM) sind bei der Diagnose eines Goodpasture-Syndroms wegweisend [57]. Eine Hautbeteiligung (v. a. Vaskulitis) spricht wiederum stark gegen diese Diagnose^9^. Auch eine kryoglobulinämische oder kryofibrinogenämische Vaskulitis ist bei Purpura, Arthralgien und nephritischem Syndrom zu bedenken [60]. Laboruntersuchungen sollten daher ein Urinsediment, eine Immunfixation (monoklonale Gammopathie), eine Hepatitisserologie und die Bestimmung der Kryoglobuline sowie Kryofibrinogen umfassen. Eine Immunglobulin-A-Vaskulitis kann durch eine Biopsie von Haut^10^ und Niere (Nachweis von IgA mittels Immunfluoreszenz) abgegrenzt werden [61]. Bei Nachweis von ANA, dsDNA-Antikörpern, Blutbildveränderungen und Hautvaskulitis ist auch ein systemischer Lupus erythematodes (SLE) zu diskutieren.

#### Infektionen.

An eine infektiöse Endokarditis (i.v.-Drogenabusus?) sollte insbesondere bei Fieber, Hautvaskulitis und Nierenbeteiligung gedacht werden. Im Zweifel sollte eine Abklärung mittels transösophagealer Echokardiographie und wiederholten Blutkulturen erfolgen.

#### Gerinnungsstörungen.

Bei schmerzhaften Hautulzerationen und -nekrosen („Angina cutis“) im Bereich der unteren Extremität ist auch eine Livedovaskulopathie (LV) zu berücksichtigen, die jedoch im Gegensatz zur CLIV *kein* entzündliches Krankheitsbild darstellt. Klinisch ist die LV auf die untere Extremität beschränkt. Bei der LV finden sich in der histologischen Untersuchung von Hautbiopsaten keine vaskulitischen Veränderungen [62]. Stattdessen bestehen v. a. intraluminale Fibrinthromben in der oberen und mittleren Dermis [62]. Arterielle oder venöse Thrombosen sind bei der CLIV selten beschrieben worden [13] und sollten daher auch an ein Antiphospholipidantikörpersyndrom (APS) denken lassen. Hierbei ist zu berücksichtigen, dass die Autoimmunserologie bei CLIV paradoxerweise auch häufig Antikörper passend zu einem APS zeigt.

### Therapie

Zur Therapie der CLIV liegen keine systematischen Untersuchungen vor. Ein Konsens besteht in der Notwendigkeit zur Beendigung des Substanzkonsums [13]. Eine suchtmedizinische Behandlung ist aufgrund der häufig fehlenden Compliance empfehlenswert. Die Therapie der CLIV richtet sich nach den Organmanifestationen. Während bei einem isolierten Hautbefall möglicherweise nur eine Lokaltherapie (z. B. Wundmanagement) notwendig sein kann, führt eine organbedrohende Manifestation häufig zum Einsatz von Glukokortikoiden [13, 15, 49]. Eine gleichzeitig bestehende Neutropenie kann jedoch die Entscheidung über eine immunsuppressive Therapie erschweren. Bei renaler Beteiligung erfolgt in vielen Fällen eine potente Immunsuppression mit CYC oder RTX [15].

## Laboranalytischer Nachweis von Kokain und Levamisol

In Blutproben kann Kokain bis zu 48 h lang nachgewiesen werden [63]. Nach Applikation einer Einzeldosis können Kokainmetaboliten im Urin bis zu 96 h lang detektiert werden [64]. Bei höheren Dosierungen und chronischem Konsum ist dies bis zu 14 Tage lang möglich [64].

Der Nachweis von Levamisol ist aufgrund der geringen Halbwertszeit im Blut und Urin (5,6 h) schwieriger [65]. Aufgrund der häufigen Beimischung von Levamisol in Kokainproben kann der Nachweis von Kokain jedoch als Surrogatmarker betrachtet werden.

Alternativ können auch Haarproben auf Kokain und Levamisol untersucht werden [66–68].

## Fazit für die Praxis

- Kokain und Levamisol können zu Erkrankungen führen, die das klinische Bild einer AAV imitieren können. CIMDL und CLIV sollten daher bei der Differenzialdiagnostik der AAV bedacht werden.
- CIMDL können durch Klinik (systemische Manifestationen), Histologie (GPA-Läsionen) und ANCA-Diagnostik (Nachweis von HLE-ANCA) von der lokalisierten GPA abgegrenzt werden.
- Die CLIV kann dem klinischen Phänotyp einer primären AAV entsprechen. Als charakteristische Befunde einer CLIV gelten nekrotische Hautveränderungen der Ohren und eine Neutropenie.
- In „therapierefraktären Fällen“ einer vermuteten AAV sollte neben Infektionen und chronischen Organschäden auch ein habitueller Kokainkonsum ausgeschlossen werden. Gerade eine „lokalisierte GPA“ mit fehlendem Ansprechen auf Glukokortikoide und potente DMARDs (RTX, CYC) ist verdächtig.

## Footnotes

1 (R)-(–)Kokain ist das einzige Diastereomer mit einem Abhängigkeitspotenzial [1].

2 Weitere Vaskulitis-Mimics haben Kötter und Reinhold-Keller [10] beschrieben.

3 In englischsprachigen Publikationen wird der Begriff „human neutrophil elastase“ (HNE) verwendet, weshalb auch von „HNE-ANCA“ gesprochen wird.

4 Die neuen Klassifikationskriterien des American College of Rheumatology (ACR) und der European Alliance of Associations for Rheumatology (EULAR) für die GPA von 2022 fordern für die Klassifikation als GPA ausdrücklich den Ausschluss von Mimics [35].

5 Bisher sind nur wenige Einzelfälle von CIMDL mit gleichzeitiger Manifestation einer systemischen Vaskulitis beschrieben worden [15, 36, 37].

6 Aus der klinischen Erfahrung ist uns der Fall einer Patientin mit Nasenseptumperforation bei Kokainkonsum bekannt, in dem anhand histologischer Befunde (fibrosiertes Gewebe der Nasenschleimhaut mit lymphoplasmazellulären Infiltraten, Plasmazellen mit Positivität für IgG bei partieller Positivität für IgG4 auf etwa der Hälfte der Plasmazellen) initial die Diagnose einer „IgG4-related midline destructive lesion“ gestellt wurde (Fall bisher nicht publiziert).

7 Eine illustrative Darstellung eines solchen Befundes findet sich bei López-Sánchez et al. [48].

8 Die Anwendung der neuen Klassifikationskriterien für die AAV (ACR/EULAR) setzt den Ausschluss von Vaskulitis-Mimics voraus [35, 55, 56].

9 Aufgrund eines gehäuften Auftretens von Goodpasture-Syndrom und primärer AAV bzw. Nachweis von ANCA kann die Abgrenzung im Einzelfall schwierig sein [58, 59].

10 Es ist zu betonen, dass die Immunglobulin-A-Vaskulitis und die kryoglobulinämische Vaskulitis im Routinepräparat (Hautbiopsie) nicht von einer leukozytoklastischen Vaskulitis abgegrenzt werden können [33].
